# The Rabl configuration limits topological entanglement of chromosomes in budding yeast

**DOI:** 10.1038/s41598-019-42967-4

**Published:** 2019-05-01

**Authors:** Maxime Pouokam, Brian Cruz, Sean Burgess, Mark R. Segal, Mariel Vazquez, Javier Arsuaga

**Affiliations:** 1 Department of Molecular and Cellular Biology, University of California, Davis, CA 95616 USA; 2 Department of Mathematics, University of California, Berkeley, CA 94720 USA; 3 Department of Epidemiology and Biostatistics, University of California San Francisco, San Francisco, CA 94143 USA; 4 Department of Mathematics, University of California, Davis, CA 95616 USA; 5 Department of Microbiology and Molecular Genetics, University of California, Davis, CA 95616 USA; 6 Department of Statistics, University of California, Davis, CA 95616 USA

**Keywords:** Computational models, Scientific data

## Abstract

The three dimensional organization of genomes remains mostly unknown due to their high degree of condensation. Biophysical studies predict that condensation promotes the topological entanglement of chromatin fibers and the inhibition of function. How organisms balance between functionally active genomes and a high degree of condensation remains to be determined. Here we hypothesize that the Rabl configuration, characterized by the attachment of centromeres and telomeres to the nuclear envelope, helps to reduce the topological entanglement of chromosomes. To test this hypothesis we developed a novel method to quantify chromosome entanglement complexity in 3D reconstructions obtained from Chromosome Conformation Capture (CCC) data. Applying this method to published data of the yeast genome, we show that computational models implementing the attachment of telomeres or centromeres alone are not sufficient to obtain the reduced entanglement complexity observed in 3D reconstructions. It is only when the centromeres and telomeres are attached to the nuclear envelope (i.e. the Rabl configuration) that the complexity of entanglement of the genome is comparable to that of the 3D reconstructions. We therefore suggest that the Rabl configuration is an essential player in the simplification of the entanglement of chromatin fibers.

## Introduction

All organisms pack their genomes in small volumes in a manner that allows for basic DNA transactions to take place^1–4^. It is, however, well known that spatial confinement promotes the topological entanglement of DNA molecules^5–16^ and that topological entanglement inhibits basic DNA transactions including gene expression and chromosome segregation^17–20^. How the eukaryotic cell balances function and topological entanglement is poorly understood. At large scales, eukaryotic chromosomes are organized into territories^21,22^ and compartments^23,24^. Both structures are present across cell types and experimental conditions^25^, with compartments been partially determined by the epigenetic state of the genome^23,26^. At the megabase-pair resolution, in an apparently self-similar fashion^24,27–29^, chromosomes are partitioned into domains and loops^30–36^ in an evolutionary preserved manner^37,38^. Several biophysical mechanisms, such as active loop extrusion^39–41^ and protein bridging^42^, and their corresponding models which include block co-polymers and random branched trees^43,44^ have been proposed to explain the possibly diverse origin of chromosomal domains and loops. Interestingly, these levels of organization are not independent of each other suggesting a sophisticated crosstalk of chromosome organization across scales^25,45^.

First described at the end of the nineteenth century^46^, the Rabl configuration is an evolutionary conserved feature of the 3D nuclear organization^47,48^. The Rabl configuration is characterized by the clustering of centromeres on one side of the nuclear envelope^49,50^ and is believed to reflect the orientation of chromosomes from the preceding mitosis^51^. Analysis of fluorescently tagged chromosomal loci in yeast revealed that, in the Rabl configuration, subtelomeric regions are positioned non-randomly near the nuclear periphery with their relative position and distance from subtelomeric regions in other chromosome arms influenced by chromosome arm length^51,52^. This analysis also revealed an axis that passes through the center of the nucleus, with centromeres clustered opposite to the nucleolus at one end of the nuclear periphery^53^. Functionally, the preservation or disruption of the Rabl configuration has been associated with chromosome segregation^54^ and DNA repair processes^55^.

Here we hypothesized that the Rabl configuration significantly reduces the incidence of chromosome entanglements during interphase. To test this hypothesis we turned to budding yeast. The three dimensional organization of the budding yeast genome is much simpler than that of the human genome, its chromosome territory structure is much less pronounced, and its overall organization is believed to be closer to equilibrium than that of the mammalian genome^56,57^. On the other hand, the budding yeast genome preserves some of the basic packaging features of higher organisms^21,53,58^ and presents very strong evidence for the Rabl configuration^49,50,59,60^. We used previously published 3D reconstructions obtained from chromosome conformation capture (CCC) data which have successfully illuminated many aspects of 3D chromosomal architecture in yeast including chromatin structure, functional compartmentalization and DNA repair^61–63^.

Quantification of the entanglement complexity between two chains is a well-defined mathematical problem when the chains are circular; in this case entanglement complexity is commonly quantified by topological invariants, including the linking number^64^. Since chromosomes are open chains (i.e. non-circular), new mathematical tools are required for quantifying their entanglement^65–67^. Here we propose a method inspired by advances in knot identification in proteins^68^. There, the knot type of the protein backbone is identified by producing ensembles of closed circular chains associated to a single protein backbone conformation. The topology of the protein is probabilistic in nature and determined by the proportion of different knots observed in the associated ensembles. We extend this approach by examining the entanglement of pairs of open chains, and by introducing the linking proportion for two open chains as a measure of their entanglement complexity. The distribution of the linking proportions associated to each pair of chromosomes in a CCC reconstruction quantifies the entanglement complexity of the reconstruction and provides a measure to compare experimentally derived reconstructions against each other and against theoretical models.

To quantify the entanglement complexity of the yeast genome, we compared the distributions of linking proportions associated with CCC reconstructions to the distributions of linking proportions associated to randomly embedded semiflexible (wormlike) chains in confinement. We found that the entanglement complexity of all reconstructions was lower than the entanglement complexity predicted by the wormlike chain model. This finding validates our approach and provides further evidence that the yeast genome is not randomly organized in the interphase nucleus^52,69–71^. We also show that the entanglement complexity of a random organization of simulated yeast chromosomes can be significantly reduced by attachments of centromeres or telomeres to the nuclear envelope; although these cannot fully account for the low entanglement complexity observed in the 3D reconstructions of the yeast genome. The absence of entanglement observed in the reconstructions is achieved only with the implementation of the Rabl configuration. We therefore suggest that the Rabl configuration is a key organizational feature that prevents the yeast chromosomes from becoming entangled.

## Data, Models and Methods

### Data

**Figure 1:**
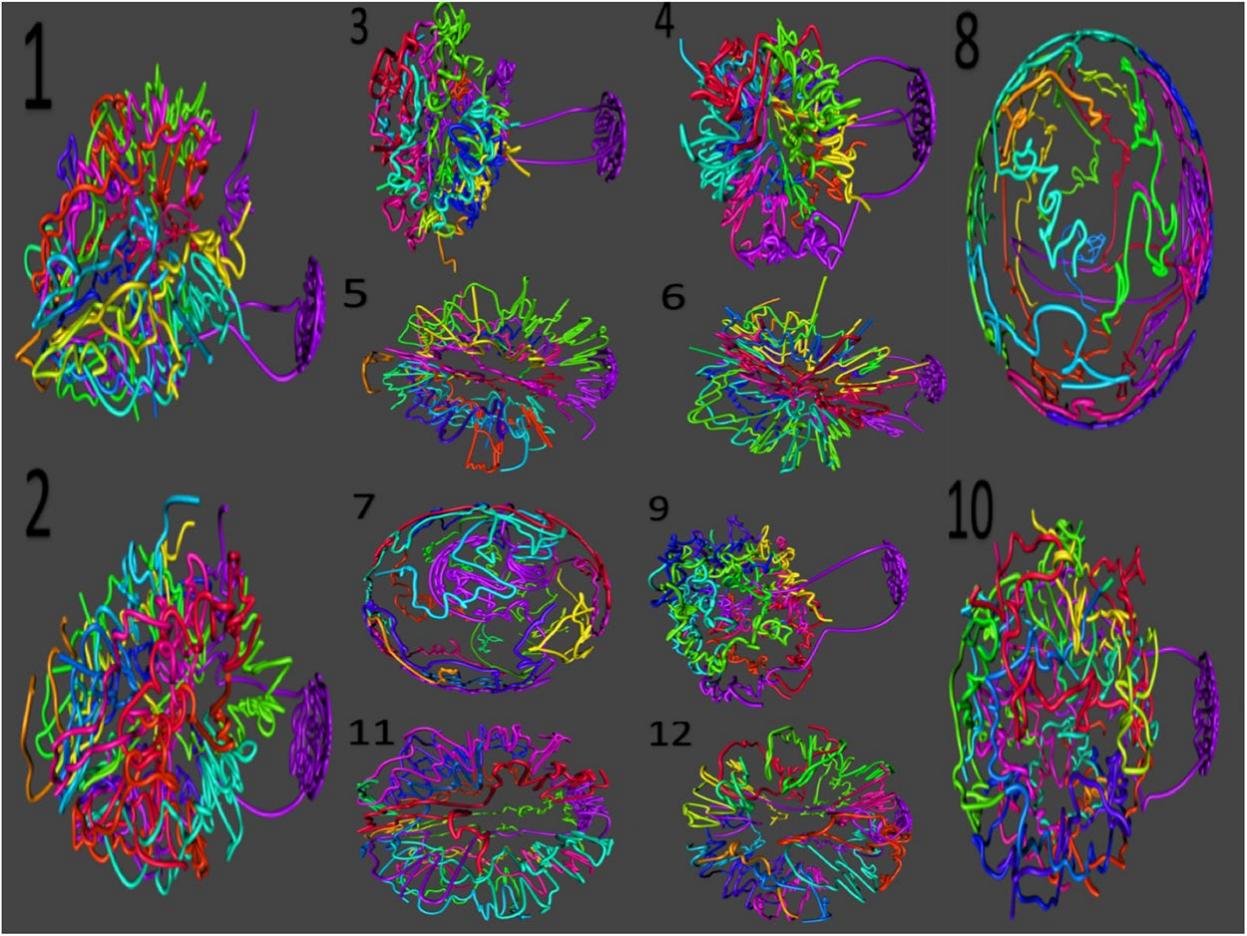
Reconstructions of the yeast genome derived from CCC data. Panel (1) shows the reconstruction using data from^62^ and Panel (2) shows a replicate of Panel (1) obtained using the same input parameters. Panels (2–12) Reconstructions obtained in^72^ using different experimental conditions and reconstruction parameters. All reconstructions are consistent with CCC data.

In previous works we generated a set of eleven 3D reconstructions of the yeast genome^72^ using data published by Duan and colleagues^62^. Reconstructions in Figure 1 were obtained using those published data. These reconstructions were the product of two consecutive restriction reactions with different enzymes, different false discovery rate (FDR) threshold^73^ at the time of significance assessment of contacts, and/or different physical distances. Reconstruction 1 was the reconstruction reported in the publication by Duan and colleagues; It was obtained from contact maps using HindIII followed by the combination of MseI and MspI (denoted by MseI $\cup$ MspI) restriction assays and a FDR threshold of 0.01. Reconstructions 2–4 were obtained by changing the FDR threshold (0.01, 0.1 and 1.0). Reconstructions 5, 6, 7 and 8 used restriction enzyme HindIII followed by MseI (Reconstruction 5), MspI (Reconstruction 6) and MseI $\cup$ MspI (reconstructions 7 and 8). Additionally, reconstructions 7 and 8 used recomputed physical distances. Reconstruction 9 used the common fragments obtained from EcoRI and HindIII (denoted by EcoRI $\cap$ HindIII) followed by MseI $\cup$ MspI; reconstruction 10 used EcoRI followed by MseI $\cap$ MspI and reconstructions 11 and 12 used EcoRI followed by MseI and MspI repectively^72^. Distance units in the reconstructions reported here coincide with those used in^72^, and are proportional to, the experimental measurements^62^.

### Quantification of the entanglement complexity of the yeast genome and classification of 3D reconstructions

**Figure 2:**
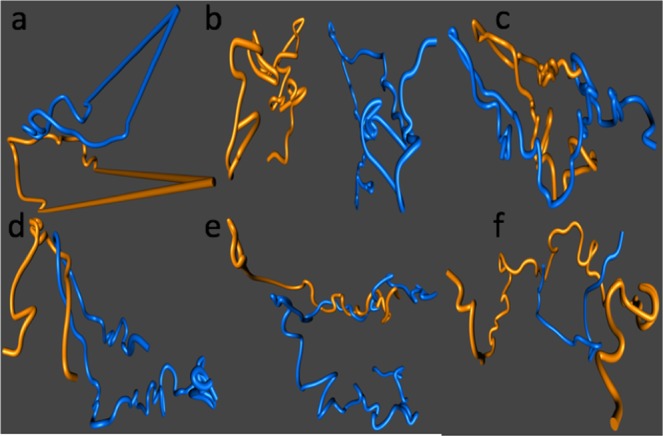
Circularization algorithm and examples of linking proportions for chromosome reconstructions from reconstructions 1, 9 and 10. (**a**) The circularization algorithm applied to two chromosome reconstructions. (**b**) Reconstructions for Chromosomes X (yellow) and XVI (blue) with $PLk=7.7 \%$ (**c**) Chromosomes X (yellow) and XIV (blue) with $PLk=80.0 \%$ (**d**) Chromosomes VI (yellow) and XIII (blue) with $PLk=85 \%$ (**e**) Chromosomes IX (yellow) and XVI (blue) from Reconstruction 9, with $PLk=51.3 \%$ (**f**) Chromosomes II (yellow) and VI (blue) from Reconstruction 10, with $PLk=53.8 \%$.

To determine the topological complexity of the yeast genome we defined a new geometrical invariant that extends the concept of linking number for open curves. We use the single stochastic closure method^68,74^ to associate an ensemble of closed trajectories to each chromosome reconstruction. For this purpose, we define a sphere *S* with its center coinciding with the center of mass of the original reconstruction (all 16 chromosomes), and radius $R > r$, where *r* is the radius of the smallest sphere containing the reconstruction (*e*.*g*. $r\approx 110$ and $R=150$). We then define a circular trajectory for each chromosome by tracing two rays connecting the telomeres of each chromosome reconstruction with a point *P* chosen at random on the surface of the sphere *S* (Fig. 2a). We obtained an ensemble of closed trajectories associated to the chromosome by repeating this process for *P*_*i*_, $i=1,\ldots ,N$. In the results reported below we used $N={10}^{3}$. To validate our method, we recomputed the linking proportion for different values of *R*, *P* and *N* and obtained consistent results. We define the linking proportion for the reconstruction of two chromosomes *l*_1_ and *l*_2_ in a given reconstruction or simulation as

$$PLk({l}_{1},{l}_{2})=\frac{1}{N}\,\sum _{k=1}^{N}\,{X}_{k}(i,j)$$

Here ${X}_{k}(i,j)=1$ if $I({C}_{1}^{i},{C}_{2}^{j})\ne 0$ and $I({C}_{1}^{i},{C}_{2}^{j})=0$ otherwise, where *I* is a topological invariant, which in our study is the linking number, of two circular trajectories ${C}_{1}^{i}$ and ${C}_{2}^{j}$ associated, through the single stochastic closure method, to the linear trajectories *l*_1_ and *l*_2_. We calculated the linking number using the double Gaussian integral form^75^ and compiled the linking proportion results in a lower triangular matrix, as illustrated in Table 1. Using the Kolmogorov-Smirnov (KS) test^76^ we determined differences between reconstructions by testing whether the distribution of entries in the tables in their vectorized form^77^ were derived from the same distribution (Section 2.3). Differences between reconstructions and simulated configurations were estimated using the Wilcoxon test (Section 2.5). In both statistical tests we assumed samples were independent and identically distributed. All p-values were corrected using FDR.

**Table 1:** The linking proportions of the 16 chromosomes in Reconstruction 1 (Fig. 1).

	I	II	III	IV	V	VI	VII	VIII	IX	X	XI	XII	XIII	XIV	XV	XVI
I	—	—	—	—	—	—	—	—	—	—	—	—	—	—	—	—
II	8.3	—	—	—	—	—	—	—	—	—	—	—	—	—	—	—
III	5.1	79.3	—	—	—	—	—	—	—	—	—	—	—	—	—	—
IV	3.9	13.6	5.6	—	—	—	—	—	—	—	—	—	—	—	—	—
V	13.7	15.7	9.2	8.6	—	—	—	—	—	—	—	—	—	—	—	—
VI	14.8	8.1	8.0	17.6	9.5	—	—	—	—	—	—	—	—	—	—	—
VII	9.8	25.9	15.3	11.8	35.6	7.4	—	—	—	—	—	—	—	—	—	—
VIII	7.7	29.4	83.9	9.4	10.7	12.9	15.0	—	—	—	—	—	—	—	—	—
IX	12.4	14.9	9.1	5.2	48.9	10.9	24.0	12.4	—	—	—	—	—	—	—	—
X	24.2	8.2	4.0	11.1	16.6	12.9	10.6	7.3	13.8	—	—	—	—	—	—	—
XI	9.2	8.5	5.7	30.2	9.0	23.5	7.5	11.9	7.5	8.9	—	—	—	—	—	—
XII	12.1	29.6	11.8	15.7	39.5	9.8	48.9	21.6	66.5	11.6	11.1	—	—	—	—	—
XIII	5.1	16.9	10.0	16.8	11.8	85.1	14.5	17.1	9.1	5.9	20.6	13.6	—	—	—	—
XIV	18.2	16.7	6.7	24.3	31.9	11.6	20.1	9.9	23.4	80.0	10.2	22.8	11.5	—	—	—
XV	10.3	13.1	4.9	25.9	14.7	17.8	12.5	9.4	14.2	70.9	11.5	20.4	14.0	45.6	—	—
XVI	8.1	23.2	7.7	26.1	11.7	13.9	15.7	21.1	11.5	7.7	20.5	20.8	42.3	12.3	11.4	—

The entries reported are given in %. The entry in row *i* and column *j* corresponds to *PLk*(*l*_*i*_, *l*_*j*_), the linking proportions of the linear chromosomes *l*_*i*_ and *l*_*j*_. Entries highlighted in red indicate a linking proportion greater than 50%.

### Simulation of Yeast Genomes

We modeled yeast chromosomes as a discrete approximation of a semiflexible chain with no torsional constrain, a configuration known as the wormlike chain^78^. Each chromosome *C* consisted of *n*_*C*_ segments $\{{e}_{1},\ldots {e}_{{n}_{C}}\}$ of equal length *l* and an energy given by

$$E({\bf{C}})=\frac{1}{2}k\,\sum _{i=1}^{{n}_{C}-1}\,{\theta }_{i}^{2}$$

where *k* is the bending rigidity constant of the 10 *nm* fiber, and *θ*_*i*_ is the exterior angle between edges **e**_*i*_ and **e**_*i*+1_. Given the experimentally estimated value for the persistence length of the yeast genome ${L}_{p}=197\pm 62\,nm$^52^, we calculated the value of the bending rigidity constant *k* and the corresponding number of discrete segments necessary to represent each Kuhn length^78^. Each chromosome realization was obtained by applying simulated annealing to a freely jointed chain that was gradually confined inside a sphere of fixed radius (*i*.*e*. the cell nucleus) and simultaneously minimized with respect to the energy of the wormlike chain. To generate Rabl configurations the same annealing algorithm was used with the additional conditions that centromeres were clustered and telomeres were tethered to variable locations near their experimentally measured locations^79,80^. The energy potential that binds telomeres and centromeres to a specific location uses the *L*^1^ norm, which allows for more movement at higher temperatures yet more restricted movement at lower temperatures, and a smoother transition during the cooling schedule (See SI for more details).

### Generation of ensembles of open chains from closed chains

To test whether our algorithm distinguishes between conformations that are in close proximity and entangled from those that are in close proximity but untangled (Section 2.2) we associated ensembles of open chains to pairs of closed chains. We generated statistically independent ensembles of closed freely jointed chains of fixed length with their center of masses separated by a fixed distance. For a pair of closed chains *C*_1_ and *C*_2_, we randomly selected a segment in each of the closed chains (*s*_1_ and *s*_2_) removed four segments consecutive to each of the selected segments *s*_*i*_, $i=1,2$ and applied the stochastic closure algorithm to compute their linking proportion. This process was repeated for different *s*_*i*_. Proportions were averaged over all sets of open chains and used as a measure of entanglement of *C*_1_ and *C*_2_.

## Results

### The circularization algorithm quantifies the entanglement complexity of 3D reconstructions

We used the linking proportion between pairs of chromosomes (*i*.*e*. open chains *l*_1_ and *l*_2_) as a measure of entanglement complexity. Although this geometrical invariant changes with chromosome length and with the distance between the center of masses of the chromosomes, it detects entanglement between two open curves and it is robust with respect to noise inherent to CCC data, a problem that has previously obscured the geometrical interpretation of CCC data^12,23,24,27,33^. We illustrate the properties of this algorithm with some examples. Figure 2 shows 3D reconstructions corresponding to some of the entries in Table 1 (Fig. 2a–c), Supplementary Table 9 (Fig. 2e) and Supplementary Table 10 (Fig. 2f). Panel *b* shows the relative positions of chromosomes X (yellow) and XVI (blue). These chromosomes are large (745 kb and 948 kb respectively); their centers of masses are far from each other (83 reconstruction units) and their linking proportion was just 7.7%. Panel *c* shows chromosomes X (yellow) and XIV (blue), with the latter of size 784 kb. They are in close proximity (with the distance between their center of masses equal to 14 reconstruction units), and are highly intermingled producing a linking proportion of 80%. Panel *d* shows chromosomes VI (yellow) and XIII (blue). Chromosome XIII is also one of the large chromosomes in the genome (924 kb) while chromosome VI is only 270 kb. The distance between their centers of mass is large (70 reconstruction units but they are highly entangled, with a linking proportion of 85%. Panel *e* shows chromosomes IX (yellow) and XVI (blue), chromosome IX is 440 kb, their centers of mass are 37 units apart and have a linking proportion of 51.3%. Panel *f* shows chromosomes II (yellow) and VI (blue), chromosome II is 813 in length and the distance between the center of mass is 24 reconstruction units. Their linking proportion is 53.8%. Results for all linking proportions for Reconstruction 1 are shown in Table 1. Tables for all other reconstructions can be found in the supplementary material. On the basis of this work, we conclude that the circularization algorithm captures the entanglement complexity for 3D reconstructions of chromosomes.

### The single stochastic closure algorithm can distinguish between entangled and unentangled open reconstructions

To test whether our method can statistically discriminate between pairs of chains that are in close proximity and entangled from those that are in close proximity but untangled, we implemented the following statistical test. First, we generated a random sample of 1,000 pairs of closed circular freely jointed chains of equal length and with centers of mass separated by a fixed distance *d*^81^. Second, we split the population into two subpopulations: those with linking number equal to zero and those with linking number different from zero. We computed their associated ensembles of open chains and linking proportions (See Section 1.4). Third, we computed and compared the average value of the linking proportions in each of the subpopulations (with or without linking number equal to zero). The sample mean of the linking proportion was 72% for entangled chains, and 57% for untangled chains. To test whether these mean values were statistically different we generated the null distribution by permuting the linking proportion values of the entangled and untangled chains. The p-value for the permutation test was ~10^−3^ (significant for *α* = 0.05). These results were further corroborated by performing the large sample independent t-test. Hence we concluded that the linking proportions can distinguish between entangled and untangled configurations that are in very close proximity.

### The single stochastic closure algorithm outperforms the linking number for open chains when comparing reconstructions

To further validate our approach we tested whether the linking proportion obtained by our method could distinguish reconstructions (obtained with different initial conditions) better than the known linking number for open chains. Based on the work in^72^, we would expect that a statistical algorithm should distinguish between reconstructions 7 and 8 from other reconstructions since they were generated using different physical distances. Tables were vectorized and compared using the K-S test (Section 1.2). Results of this comparison are shown in Table 2. The rows and columns in the table correspond to different reconstructions and the entries show the p-value associated to the linking number for open chains (first two rows) and the stochastic closure method proposed here (second two rows). Our results clearly show that the stochastic closure method can distinguish all of the reconstructions from reconstructions 7 and 8 (except for reconstruction 11). The linking number for open chains, on the other hand, fail to distinguish eleven of them. Based on these results we conclude that the stochastic closure algorithm outperforms other standard methods to measure entanglement of open curves obtained through the CCC data analyzed here.

**Table 2:** Table of p-values comparing reconstructions 7 and 8 (obtained using the recomputed physical distances) with other reconstructions.

	1	2	3	4	5	6	7	8	9	10	11	12
7	0.008	0.085	0.069	0.039	0.035	0.039	—	—	0.035	0.004	0.794	0.465
8	0.105	0.131	0.043	0.057	0.084	0.105	—	—	0.003	0.003	0.159	0.105
7	0.003	0.015	0.012	0.005	0.015	0.018	—	—	0.00	0.00	0.072	0.036
8	0.00	0.00	0.00	0.00	0.015	0.014	—	—	0.00	0.00	0.17	0.036

The top two rows show the p-values obtained using the K-S test for the linking number defined for open (non-circular) chains. The bottom two rows show the p-values obtained using the linking proportion. The p-values were adjusted using False Discovery Rate (FDR) for multiple testing for the 12 reconstructions.

### The entanglement complexity of the yeast genome is lower than predicted by the wormlike chain model

**Figure 3:**
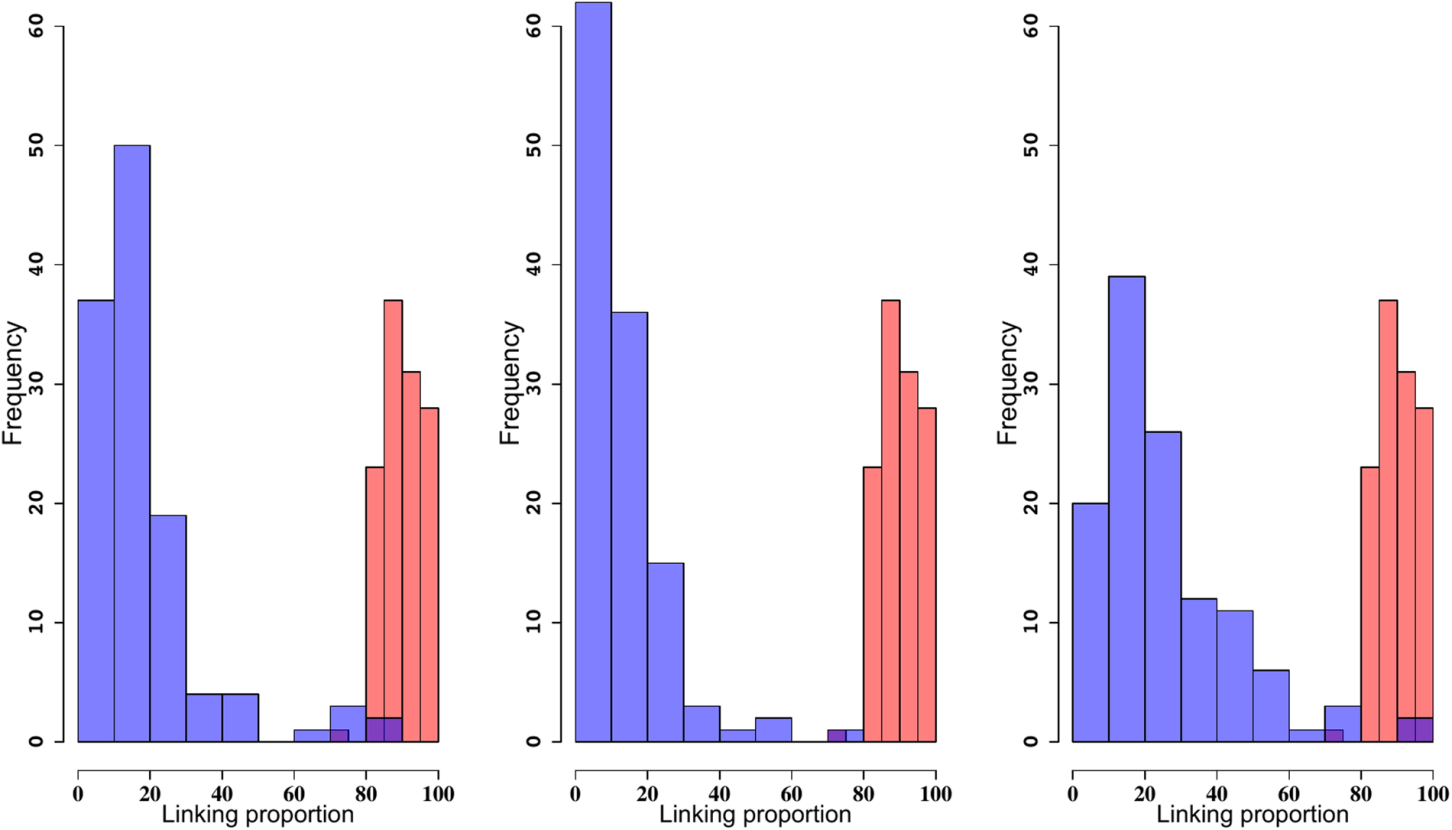
Wormlike chains have a larger entanglement complexity than reconstructions. Frequency for the linking proportions of reconstructions 1, 8 and 10 and for randomly embedded wormlike chains. The distribution colored in blue corresponds to the reconstructions and the distribution colored in red corresponds to the wormlike chains. Purple indicates overlapping values.

We compared the observed linking proportion distribution in the reconstructions with those obtained using Monte-Carlo simulations of random embeddings of wormlike chains confined to a spherical volume. The mean, median and range for the distribution of linking proportions are shown in Table 3. Figure 3 shows the histograms representing the frequency of the linking proportions of the CCC reconstructions (blue) and of wormlike chains obtained by computer simulations (red). All the means were significantly smaller than that estimated for the wormlike chain (*μ*_*wlc*_ = 81%). These results clearly show that the entanglement complexity predicted by the wormlike chains is much larger than that of the reconstructions. This finding is consistent with biophysical results of the linking of randomly embedded chains in confined volumes^82^ and it provides further evidence that the yeast genome is not randomly organized. We also illustrate this result in Fig. 3, where the distribution of linking proportions for three representative reconstructions. Reconstruction 1 (published in^62^), Reconstruction 8 and 10 with the lowest and largest mean/median of the linking proportion values respectively.

**Table 3:** Linking proportion mean, median and range values for all reconstructions reflect the simulated Rabl configuration.

Reconstructions	1	2	3	4	5	6	7	8	9	10	12	Rabl
mean	18.2	18.4	18.4	19.0	14.0	16.1	14.4	12.7	21.6	25.6	14.0	24.1
median	12.7	13.1	14.35	14.2	11.5	12.1	10.8	9.5	17.4	20.2	9.9	15.6
min	3.9	3.1	3.6	2.1	3.30	2.8	1.4	2.2	3.2	4.7	3.2	2.2
max	85.1	93.8	91.5	83.5	73.7	91.0	99.3	71.3	93.5	92.8	90.5	94.6

Each column corresponds to one reconstruction.

### The Rabl configuration of the yeast genome helps explain the observed reduced chromosome entanglement

The main genomic organizational features observed at the level of resolution of chromosome arms and territories are the clustering of centromeres and telomeres in the Rabl configuration. We therefore posit that the distinct Rabl configuration plays a key role in preventing the entanglement of chromosomes. To test this hypothesis we performed three different simulation studies: (*i*) with centromeres clustered near the nuclear envelope and free telomeres; (*ii*) with only telomeres tethered near the nuclear envelope; and (*iii*) with both centromeres and telomeres fixed, resembling the Rabl configuration.

**Figure 4:**
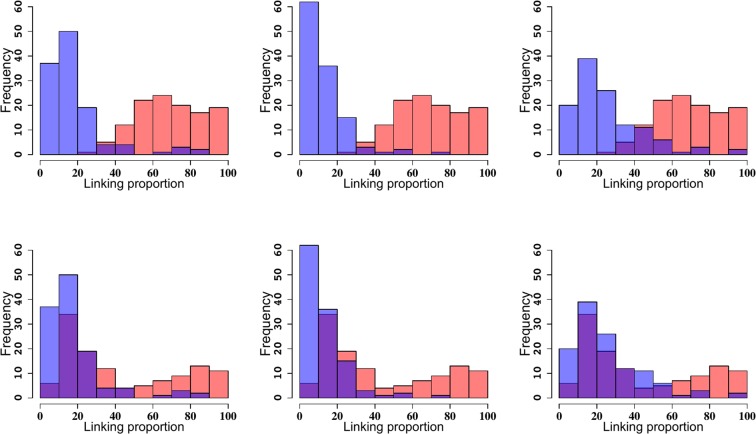
Histograms of linking proportions for for reconstructions 1, 8 and 10 (left to right) and for randomly embedded wormlike chains with only centromeres (top three histograms) or only telomeres (bottom three histograms) attached to the nuclear envelope. The blue histogram corresponds to the reconstructions and the red to the wormlike chains. Purple indicate overlapping values. Both models are characterized by entanglement complexity values larger than the values observed in reconstructions.

The top three panels in Fig. 4 show the distribution of linking proportions corresponding to simulation *(i)*. The distributions of linking proportions corresponding to the selected reconstructions in (blue) still show much lower linking proportion than when only centromeres are clustered at the nuclear envelope (red) (*μ*_*cent*_ = 70 ± 20%). The lower three panels show the results for simulation *(ii)*, in which only telomeres are attached near the nuclear envelope^52,70^. Interestingly, in case *(ii)*, the distribution of linking number frequencies (*μ*_*tel*_ = 60 ± 30%) was closer to that of the reconstructions and much simpler than those of the randomly embedded wormlike chain and those in which only centromeres were attached (Fig. 4, top three panels). These results clearly show that these mechanisms are not sufficient to reduce the entanglement complexity to levels similar to those observed in the reconstruction data.

**Figure 5:**
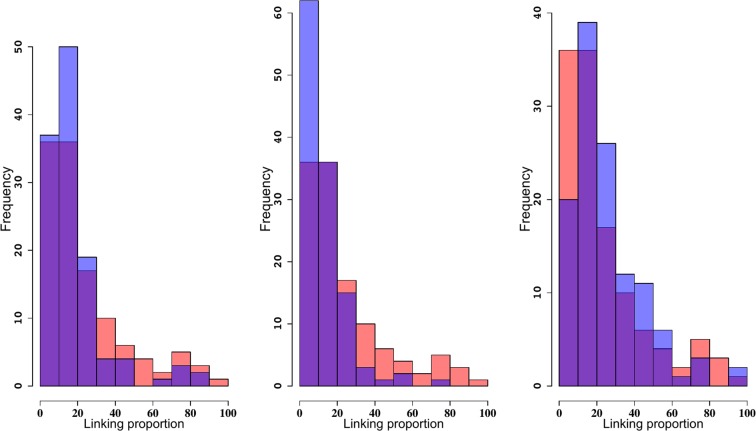
Histograms of the linking proportions for reconstructions 1, 8 and 10 (left to right) and for simulated Rabl configurations (both centromeres and telomeres attached to the nuclear envelope). Blue corresponds to reconstructions and red to simulated data. The histograms overlap showing that the Rabl configurations decreases the entanglement of the genome to levels comparable to those observed in the experimental data.

The situation was different when we implemented the Rabl configuration in simulation (iii). In this case the topological complexity was reduced to levels comparable to those observed in the reconstructions and the mean of the distribution of linking numbers was *μ*_*Rabl*_ = 24 ± 21% a value much closer to those observed in the reconstructions (Fig. 5). In fact there were no significant differences between the simulated configurations and Reconstructions 1 and 10 ($p=0.062$ and $p=0.062$). Interestingly, we still found significant differences between reconstruction 8 and the simulated models. Inspection of this reconstruction revealed that chromatin fibers have more interactions with the nuclear envelope than our proposed Rabl configuration. This reconstruction suggests a mechanism of entanglement simplification driven by the frequent attachment of chromatin fibers to the nuclear envelope^83^. Based on these results we suggest that the Rabl configuration is a regulator of the three dimensional organization of genomes that prevents entanglement of chromosome fibers.

## Conclusion and Discussion

The three dimensional organization of genomes is essential for the correct functioning of the cell. Confinement of DNA fibers, however, promotes entanglement as evidenced experimentally by DNA knots and links observed in some viruses^5,84^ and in the mitochondrial DNA of kinetoplastids (reviewed in^85^), and by multiple theoretical studies^6,7,9–11,13,86,87^. Organisms have evolved mechanisms to regulate DNA entanglement. Most notable is the presence of topoisomerases and site specific recombinases, enzymes that regulate the topology of genomes and that are known to unknot and unlink DNA^88–90^. There is evidence however that the cell has evolved other mechanisms to regulate the entanglement of genomes. For instance, at large scales, the eukaryotic chromosome is confined into territory (reviewed in^22^) and below the megabase scale, genomes are partitioned into domains and loops, an organizational feature that is preserved from bacteria^37,38^ to humans^30–34,36,42,91,92^.

On the basis of the work reported here, we propose that the Rabl configuration, an organizational feature that is also preserved through multiple species in evolution, provides another mechanism for topology simplification. We tested this hypothesis using published CCC data and developed a new method in statistical topology to estimate the topological entanglement of genomes. Our method for estimating entanglement has some advantages over the standard linking number for open chains. First, it detects entanglement of curves better than the standard linking number for open curves when analyzing 3D reconstructions and second it can be extended by using topological invariants finer than the linking number. Whether the former is a general property or not remains to be determined. Our results showed that the entanglement complexity of CCC reconstructions of the yeast genome is lower than the entanglement complexity of free randomly embedded chains and chains in which centromeres and telomeres had been tethered to the nuclear envelope; only the implementation of the Rabl configuration yielded an entanglement complexity comparable to that of the CCC reconstructions. These results suggest that the Rabl configuration is a regulator of the entanglement complexity of the genome. Note however that this finding does not exclude the possibility of other mechanisms such as the tethering of other regions of the genome to the nuclear envelope^20,83^. Our method and conclusions are limited by the fact that chromosomes are highly dynamic, specially as the cell goes through the cell cycle. This limitation opens the door to new inquiries on topology regulation during the cell cycle.

## Supplementary information

Supplementary Data

## Data Availability

The datasets generated during and/or analysed during the current study are available from the corresponding author on reasonable request.
