# Supplementary material for: The Rabl configuration limits topological entanglement of chromosomes in budding yeast
Source: Sci Rep. 2019 May 1;9:6795. doi: 10.1038/s41598-019-42967-4 (PMC6494875; doi:10.1038/s41598-019-42967-4)
Supplement: Supplementary file 1 — Supplementary Data [file 41598_2019_42967_MOESM1_ESM.pdf]

The Rabl configuration limits topological entanglement of chromosomes in budding yeast

Maxime Pouokam[1], Brian Cruz[2], Sean Burgess[1], Mark Segal [3], Mariel Vazquez[4,5], Javier Arsuaga[1,4]

[1]Department of Molecular and Cellular Biology, University of California, Davis, CA 95616
[2] Department of Mathematics, University of California, Berkeley, CA 94720
[3]Department of Biostatistics, University of California San Francisco, San Francisco CA 94143
[4]Department of Mathematics, University of California, Davis, CA 95616
[5]Department of Microbiology and Molecular Genetics, University of California, Davis, CA 95616
⋆ jarsuaga@ucdavis.edu

1. Supplementary Information

Table S1. Chromosome linking proportion for reconstruction 5. The highlighted number in red indicated the linking proportion that are greater than 50 %.

	I	II	III	IV	V	VI	VII	VIII	IX	X	XI	XII	XIII	XIV	XV	XVI
I	-	-	-	-	-	-	-	-	-	-	-	-	-	-	-	-
II	9.5	-	-	-	-	-	-	-	-	-	-	-	-	-	-	-
III	4.8	5.8	-	-	-	-	-	-	-	-	-	-	-	-	-	-
IV	6.5	39.4	5.3	-	-	-	-	-	-	-	-	-	-	-	-	-
V	8.1	17.8	11.7	19.5	-	-	-	-	-	-	-	-	-	-	-	-
VI	9.7	5.1	7.5	5.3	7.3	-	-	-	-	-	-	-	-	-	-	-
VII	4.4	10.1	12.1	8.5	13.6	8.8	-	-	-	-	-	-	-	-	-	-
VIII	13.6	14.0	12.3	13.4	16.9	14.1	15.8	-	-	-	-	-	-	-	-	-
IX	4.6	6.4	10.8	9.6	10.7	11.5	29.2	15.5	-	-	-	-	-	-	-	-
X	3.3	6.6	8.7	6.1	23.2	6.3	8.2	5.9	6.4	-	-	-	-	-	-	-
XI	10.3	12.5	8.2	10.5	9.7	15.6	13.1	49.5	16.4	5.6	-	-	-	-	-	-
XII	5.8	19.0	16.2	22.9	38.3	8.6	20.0	16.7	11.9	23.9	13.6	-	-	-	-	-
XIII	14.3	22.3	8.4	18.6	15.2	9.8	12.5	25.4	9.2	4.7	22.1	17.8	-	-	-	-
XIV	5.6	17.6	10.9	18.7	33.1	7.6	11.2	9.4	7.2	20.9	8.8	41.5	11.7	-		
XV	4.0	9.1	14.7	10.3	18.4	7.9	73.7	11.5	13.9	24.6	9.4	24.7	8.3	18.4	-	-
XVI	8.4	10.3	10.0	9.0	9.0	13.9	21.1	26.9	17.9	6.1	24.7	13.3	15.2	7.0	12.8	-

Table S2. Chromosome linking proportion for reconstruction 2. The highlighted number in red indicated the linking proportion that are greater than 50 %.

	I	II	III	IV	V	VI	VII	VIII	IX	X	XI	XII	XIII	XIV	XV	XVI
I	-	-	-	-	-	-	-	-	-	-	-	-	-	-	-	-
II	9.5	-	-	-	-	-	-	-	-	-	-	-	-		-	-
III	7.6	21.7	-	-	-	-	-	-	-	-	-	-	-	-	-	-
IV	6.3	26.8	7.9	-	-	-	-	-	-	-	-	-	-	-	-	-
V	11.0	15.2	13.3	13.3	-	-	-	-	-	-	-	-	-	-	-	-
VI	4.7	15.7	9.1	8.1	7.8	-	-	-	-	-	-	-	-	-	-	-
VII	7.1	18.0	5.5	27.0	7.9	5.2	-	-	-	-	-	-	-	-	-	-
VIII	25.0	16.0	6.9	15.1	18.7	5.5	12.3	-	-	-	-	-	-	-	-	-
IX	24.1	15.9	8.2	10.8	18.9	3.1	12.0	55.9	-	-	-	-	-	-	-	-
X	6.6	27.4	14.2	23.3	15.0	19.5	10.5	10.1	10.5	-	-	-	-	-	-	-
XI	4.9	35.6	6.8	20.0	10.0	25.3	17.9	9.2	4.3	15.5	-	-	-	-	-	-
XII	7.6	28.1	7.3	69.9	8.7	11.0	57.3	17.9	9.8	93.8	16.1	-	-	-	-	-
XIII	7.5	62.1	10.2	29.3	10.4	57.0	14.9	10.9	7.5	25.2	83.4	85.7	-	-	-	-
XIV	12.1	12.0	8.0	13.7	13.5	5.0	12.7	20.3	19.9	7.7	7.7	14.5	10.1	-	-	-
XV	14.5	18.0	3.9	19.5	11.5	6.0	24.9	21.6	18.1	9.7	10.9	26.4	10.2	63.4	-	-
XVI	11.7	17.1	4.2	19.6	9.6	6.9	36.9	17.1	17.2	11.6	12.9	31.7	10.8	31.0	45.4	-

Table S3. Chromosome linking proportion for reconstruction 3. The highlighted number in red indicated the linking proportion that are greater than 50 %.

	I	II	III	IV	V	VI	VII	VIII	IX	X	XI	XII	XIII	XIV	XV	XVI
I	-	-	-	-	-	-	-	-	-	-	-	-	-	-	-	-
II	10.3	-	-	-	-	-	-	-	-	-	-	-	-	-	-	-
III	10.4	15.1	-	-	-	-	-	-	-	-	-	-	-	-	-	-
IV	6.4	16.3	6.8	-	-	-	-	-	-	-	-	-	-	-	-	-
V	9.1	31.3	6.1	25.8	-	-	-	-	-	-	-	-	-	-	-	-
VI	6.4	4.7	8.2	8.8	6.4	-	-	-	-	-	-		-	-	-	-
VII	10.7	22.7	70.5	55.0	12.5	12.9	-	-	-	-	-	-	-	-	-	-
VIII	7.5	12.0	3.6	14.5	17.0	9.8	14.1	-	-	-	-	-	-	-	-	-
IX	9.2	36.3	5.8	8.0	28.2	3.9	12.9	9.8	-	-	-	-	-	-	-	-
X	9.2	25.5	32.6	14.0	11.6	5.8	41.7	8.3	12.0	-	-	-	-	-	-	-
XI	6.6	23.6	5.3	16.6	55.4	4.0	12.3	15.3	23.1	9.1	-	-	-	-	-	-
XII	6.3	31.7	5.7	37.1	20.1	6.2	38.6	21.3	15.5	16.1	26.8	-	-	-	-	-
XIII	3.7	15.1	3.9	33.4	20.6	7.9	21.3	23.3	10.8	10.8	15.2	30.3	-	-	-	-
XIV	12.1	12.5	91.5	11.5	13.0	27.7	22.4	17.1	11.5	11.9	14.2	15.8	19.8	-	-	-
XV	6.5	14.6	8.9	13.5	18.6	11.9	24.5	37.3	11.5	12.9	15.2	25.7	53.9	28.3	-	-
XVI	5.9	36.2	6.1	17.4	29.3	5.3	22.6	20.6	17.2	19.1	51.9	48.6	25.6	16.1	20.9	-

Table S4. Chromosome linking proportion for reconstruction 4. The highlighted number in red indicated the linking proportion that are greater than 50 %.

	I	II	III	IV	V	VI	VII	VIII	IX	X	XI	XII	XIII	XIV	XV	XVI
I	-	-	-	-	-	-	-	-	-	-	-	-	-	-	-	-
II	5.4	-	-	-	-	-	-	-	-	-	-	-	-	-	-	-
III	2.1	11.8	-	-	-	-	-	-	-	-	-	-	-	-	-	-
IV	7.4	24.9	6.4	-	-	-	-	-	-	-	-	-	-	-	-	-
V	7.9	22.0	21.3	14.0	-	-	-	-	-	-	-	-	-	-	-	-
VI	17.2	9.2	4.6	18.8	7.1	-	-	-	-	-	-	-	-	-	-	-
VII	13.0	11.9	4.3	18.6	11.4	7.5	-	-	-	-	-	-	-	-	-	-
VIII	12.6	12.9	10.4	14.1	15.9	40.7	20.5	-	-	-	-	-	-	-	-	-
IX	9.6	12.6	10.4	8.6	26.4	11.2	15.6	16.3	-	-	-	-	-	-	-	-
X	6.9	10.0	10.3	8.0	76.3	10.0	9.7	8.8	78.0	-	-	-	-	-	-	-
XI	7.4	27.8	10.1	18.6	12.8	17.2	7.2	25.0	12.5	8.0	-	-	-	-	-	-
XII	15.5	62.2	6.8	29.4	25.2	22.4	31.9	42.4	23.4	14.3	66.5	-	-	-	-	-
XIII	6.6	13.8	9.0	15.2	30.1	4.8	16.8	8.9	27.7	35.0	10.6	24.0	-	-	-	-
XIV	10.7	13.9	6.3	13.8	18.2	6.5	16.8	15.8	65.7	83.5	6.5	26.1	34.9	-	-	-
XV	11.2	18.0	4.8	80.0	13.1	22.4	24.5	21.4	12.4	9.1	14.5	31.6	12.4	14.3	-	-
XVI	5.9	46.3	15.8	21.2	36.8	7.2	10.6	16.5	17.3	17.2	16.9	30.7	20.0	14.4	14.0	-

Table S5. Chromosome linking proportion for reconstruction 6. The highlighted number in red indicated the linking proportion that are greater than 50 %.

	I	II	III	IV	V	VI	VII	VIII	IX	X	XI	XII	XIII	XIV	XV	XVI
I	-	-	-	-	-	-	-	-	-	-	-	-	-	-	-	-
II	8.5	-	-	-	-	-	-	-	-	-	-	-	-	-	-	-
III	14.0	17.1	-	-	-	-	-	-	-	-	-	-	-	-	-	-
IV	2.8	7.8	6.6	-	-	-	-	-	-	-	-	-	-	-	-	-
V	10.7	64.1	26.2	9.6	-	-	-	-	-	-	-	-	-	-	-	-
VI	6.9	8.8	11.0	10.4	10.7	-	-	-	-	-	-	-	-	-	-	-
VII	4.4	13.0	8.1	90.0	9.0	16.4	-	-	-	-	-	-	-	-	-	-
VIII	12.1	14.3	10.9	13.9	14.6	10.7	10.4	-	-	-	-	-	-	-	-	-
IX	12.1	21.2	24.5	7.8	26.1	24.2	13.0	13.5	-	-	-	-	-	-	-	-
X	5.2	7.2	3.8	19.7	7.7	10.9	10.5	20.8	6.3	-	-	-	-	-	-	-
XI	5.4	21.6	7.8	7.9	19.9	5.3	7.0	12.6	12.4	6.8	-	-	-	-	-	-
XII	8.4	35.6	15.0	14.5	29.1	15.4	16.4	31.8	22.0	15.2	48.7	-	-	-		-
XIII	6.5	13.4	6.3	22.0	11.8	9.1	22.1	23.2	6.8	66.3	14.0	27.8	-	-	-	-
XIV	7.4	26.0	17.6	12.1	21.6	23.5	42.0	12.3	31.2	7.0	11.6	22.7	20.9	-	-	-
XV	4.2	15.5	8.0	9.5	15.5	12.3	19.6	7.5	16.2	5.8	8.9	11.9	9.9	91.0	-	-
XVI	5.9	14.7	7.1	9.3	8.7	3.8	6.2	28.2	8.6	12.0	18.7	22.8	18.0	9.6	6.8	-

Table S6. Chromosome linking proportion for reconstruction 7. The highlighted number in red indicated the linking proportion that are greater than 50 %.

	I	II	III	IV	V	VI	VII	VIII	IX	X	XI	XII	XIII	XIV	XV	XVI
I	-	-	-	-	-	-	-	-	-	-	-	-	-	-	-	-
II	7.8	-	-	-	-	-	-	-	-	-	-	-	-	-	-	-
III	1.9	8.2	-	-	-	-	-	-	-	-	-	-	-	-	-	-
IV	5.7	25.0	9.6	-	-	-	-	-	-	-	-	-	-	-	-	-
V	4.9	13.2	3.2	17.5	-	-	-	-	-	-	-	-	-	-	-	-
VI	1.4	2.7	2.8	8.2	2.1	-	-	-	-	-	-	-	-	-	-	-
VII	15.7	16.5	3.6	14.6	14.6	3.7	-	-	-	-	-	-	-	-	-	-
VIII	15.4	13.5	4.2	11.4	10.4	2.0	48.7	-	-	-	-	-	-	-	-	-
IX	7.2	5.1	1.6	6.4	3.4	1.8	4.5	3.0	-	-	-	-	-	-	-	-
X	8.5	10.2	3.8	12.9	37.9	2.1	15.9	15.9	4.3	-	-	-	-	-	-	-
XI	40.2	18.5	3.3	11.7	8.8	2.3	22.7	20.9	5.8	10.3	-	-	-	-	-	-
XII	24.4	25.1	6.8	43.6	23.4	4.6	35.0	46.9	11.2	20.1	55.6	-	-	-	-	-
XIII	7.4	29.9	6.4	16.9	11.6	2.0	11.9	10.6	4.1	11.2	10.9	18.9	-	-	-	-
XIV	12.4	13.4	3.3	17.5	10.5	2.8	30.0	10.8	17.8	9.7	14.4	32.7	9.6	-	-	-
XV	10.0	26.9	4.8	20.0	17.6	4.0	45.1	24.0	4.5	16.4	19.0	58.8	99.3	11.5	-	-
XVI	7.3	9.9	3.1	12.6	19.2	4.4	19.0	9.8	3.2	14.9	10.7	17.6	9.0	17.6	17.5	-

Table S7. Chromosome linking proportion for reconstruction 8. The highlighted number in red indicated the linking proportion that are greater than 50 %.

	I	II	III	IV	V	VI	VII	VIII	IX	X	XI	XII	XIII	XIV	XV	XVI
I	-	-	-	-	-	-	-	-	-	-	-	-	-	-	-	-
II	5.8	-	-	-	-	-	-	-	-	-	-	-	-	-	-	-
III	3.6	3.8	-	-	-	-	-	-	-	-	-	-	-	-	-	-
IV	8.7	36.0	5.8	-	-	-	-	-	-	-	-	-	-	-	-	-
V	14.9	7.5	3.4	11.1	-	-	-	-	-	-	-	-	-	-	-	-
VI	3.3	15.7	2.5	15.0	3.7	-	-	-	-	-	-	-	-	-	-	-
VII	23.3	10.6	4.6	17.5	23.1	6.7	-	-	-	-	-	-	-	-	-	-
VIII	10.0	10.1	3.2	71.3	14.1	5.5	20.3	-	-	-	-	-	-	-	-	-
IX	2.3	12.2	3.9	16.0	4.3	5.0	5.8	5.6	-	-	-	-	-	-	-	-
X	6.6	6.0	3.6	7.3	10.9	3.0	7.7	4.6	5.2	-	-	-	-	-	-	-
XI	7.5	8.7	2.2	11.8	10.6	4.7	9.7	15.4	5.3	8.7	-	-	-	-	-	-
XII	8.7	17.4	6.3	21.4	13.8	8.7	15.2	12.0	27.4	52.0	23.8	-	-	-	-	-
XIII	5.1	3.7	5.3	6.7	4.2	2.2	5.8	4.5	4.9	8.7	4.8	13.6	-	-	-	-
XIV	7.1	12.7	4.5	15.6	22.2	5.6	21.8	24.9	7.4	14.1	26.0	28.1	8.7	-	-	-
XV	9.3	14.7	7.5	22.6	11.6	7.7	13.3	13.7	11.7	16.3	21.3	45.7	10.4	19.3	-	-
XVI	6.4	31.4	4.8	50.6	11.9	14.2	14.5	13.7	12.4	8.3	17.0	32.4	5.7	21.7	30.0	-

Table S8. Chromosome linking proportion for reconstruction 9. The highlighted number in red indicated the linking proportion that are greater than 50 %.

	I	II	III	IV	V	VI	VII	VIII	IX	X	XI	XII	XIII	XIV	XV	XVI
I	-	-	-	-	-	-	-	-	-	-	-	-	-	-	-	-
II	12.7	-	-	-	-	-	-	-	-		-	-	-	-	-	-
III	8.4	14.3	-	-	-	-	-	-	-	-	-	-	-	-	-	-
IV	6.0	20.9	10.6	-	-	-	-	-	-	-	-	-	-	-	-	-
V	5.4	27.5	11.6	27.5	-	-	-	-	-	-	-	-	-	-	-	-
VI	4.7	8.5	4.2	14.2	10.0	-	-	-	-	-	-	-	-	-	-	-
VII	4.1	16.7	6.7	68.8	17.9	5.2	-	-	-	-	-	-	-	-	-	-
VIII	5.0	12.9	6.5	24.8	13.5	5.4	83.3	-	-	-	-	-	-	-	-	-
IX	7.4	17.2	17.5	14.6	14.1	7.1	14.1	20.2	-	-	-	-	-	-	-	-
X	3.2	14.7	8.3	43.8	34.5	12.2	32.8	13.3	9.1	-	-	-	-	-	-	-
XI	6.3	20.1	9.7	35.1	31.8	45.6	20.8	17.5	12.9	26.8	-	-	-	-	-	-
XII	7.3	33.0	11.5	45.6	51.8	5.9	83.2	93.5	20.9	22.3	18.4	-	-	-	-	-
XIII	8.8	32.6	59.4	21.5	22.8	7.8	17.4	23.9	30.6	16.8	18.9	23.5	-	-	-	-
XIV	11.4	53.6	19.7	24.2	27.1	10.5	25.7	43.7	26.1	21.1	22.1	38.1	64.8	-	-	-
XV	5.2	22.8	20.2	17.4	16.2	5.2	15.5	18.0	31.2	11.5	10.2	30.8	25.4	23.9	-	-
XVI	5.8	14.4	20.6	15.3	13.0	5.5	15.6	30.4	51.3	13.0	13.2	23.9	22.0	20.9	47.6	-

Table S9. Chromosome linking proportion for reconstruction 10. The highlighted number in red indicated the linking proportion that are greater than 50 %.

	I	II	III	IV	V	VI	VII	VIII	IX	X	XI	XII	XIII	XIV	XV	XVI
I	-	-	-	-	-	-	-	-	-	-	-	-	-	-	-	-
II	5.8	-	-	-	-	-	-	-	-	-	-	-	-	-	-	-
III	4.9	9.9	-	-	-	-	-	-	-	-	-	-	-	-	-	-
IV	5.8	41.8	8.1	-	-	-	-	-	-	-	-	-	-	-	-	-
V	6.3	37.8	7.2	19.1	-	-	-	-	-	-	-	-	-	-	-	-
VI	7.3	53.8	17.2	92.8	16.5	-	-	-	-	-	-	-	-	-	-	-
VII	21.6	18.8	13.1	20.1	15.7	19.3	-	-	-	-	-	-	-	-	-	-
VIII	6.4	21.5	6.6	42.6	26	9.4	23	-	-	-	-	-	-	-	-	-
IX	4.7	20.9	11.3	10.4	23.7	14.3	17.7	12.7	-	-	-	-	-	-	-	-
X	8.9	14.5	7	18.9	13.8	9.8	49.4	20.3	14.3	-	-	-	-	-	-	-
XI	25.1	11.7	10.4	15.3	15.6	10.6	27.9	17.3	44.4	41.1	-	-	-	-	-	-
XII	19.9	31.8	11.2	30.7	17.4	21.5	48.1	55.4	17.2	21.9	18.6	-	-	-	-	-
XIII	9.6	26.3	10	92.1	22.2	21.4	30.2	37.7	22.6	32.1	33.2	75.2	-	-	-	-
XIV	9.2	19.2	8.3	56.7	44.7	10.6	26.5	53.8	18.9	34.9	29.1	47	70.1	-	-	-
XV	9.6	31.2	44.5	24.9	35.1	40.8	27.5	25	53.2	16.9	19.7	35.3	25	23	-	-
XVI	13.2	19.4	12.7	19.2	16.5	20.4	56.8	19.5	16	60.5	76.5	35.9	23.6	45.9	26.2	

Table S10. Chromosome linking proportion for reconstruction 11. The highlighted number in red indicated the linking proportion that are greater than 50%.

	I	II	III	IV	V	VI	VII	VIII	IX	X	XI	XII	XIII	XIV	XV	XVI
I	-	-	-	-	-	-	-	-	-	-	-	-	-	-	-	-
II	9.5	-	-	-	-	-	-	-	-	-	-	-	-	-	-	-
III	4.8	5.8	-	-	-	-	-	-	-	-	-	-	-	-	-	-
IV	6.5	39.4	5.3	-	-	-	-	-	-	-	-	-	-	-	-	-
V	8.1	17.8	11.7	19.5	-	-	-	-	-	-	-	-	-	-	-	-
VI	9.7	5.1	7.5	5.3	7.3	-	-	-	-	-	-	-	-	-	-	-
VII	4.4	10.1	12.1	8.5	13.6	8.8	-	-	-	-	-	-	-	-	-	-
VIII	13.6	14.0	12.3	13.4	16.9	14.1	15.8	-	-	-	-	-	-	-	-	-
IX	4.6	6.4	10.8	9.6	10.7	11.5	29.2	15.5	-	-	-	-	-	-	-	-
X	3.3	6.6	8.7	6.1	23.2	6.3	8.2	5.9	6.4	-	-	-	-	-	-	-
XI	10.3	12.5	8.2	10.5	9.7	15.6	13.1	49.5	16.4	5.6	-	-	-	-	-	-
XII	5.8	19.0	16.2	22.9	38.3	8.6	20.0	16.7	11.9	23.9	13.6	-	-	-	-	-
XIII	14.3	22.3	8.4	18.6	15.2	9.8	12.5	25.4	9.2	4.7	22.1	17.8	-	-	-	-
XIV	5.6	17.6	10.9	18.7	33.1	7.6	11.2	9.4	7.2	20.9	8.8	41.5	11.7	-		
XV	4.0	9.1	14.7	10.3	18.4	7.9	73.7	11.5	13.9	24.6	9.4	24.7	8.3	18.4	-	-
XVI	8.4	10.3	10.0	9.0	9.0	13.9	21.1	26.9	17.9	6.1	24.7	13.3	15.2	7.0	12.8	-

Table S11. Chromosome linking proportion for reconstruction 12. The highlighted number in red indicated the linking proportion that are greater than 50%.

	I	II	III	IV	V	VI	VII	VIII	IX	X	XI	XII	XIII	XIV	XV	XVI
I	-	-	-	-	-	-	-	-	-	-	-	-	-	-	-	-
II	7.2	-	-	-	-	-	-	-	-	-	-	-	-	-	-	-
III	6.4	24.3	-	-	-	-	-	-	-	-	-	-	-	-	-	-
IV	5.4	17.9	11.1	-	-	-	-	-	-	-	-	-	-	-	-	-
V	7.1	8.0	7.7	11.8	-	-	-	-	-	-	-	-	-	-	-	-
VI	2.4	6.8	8.4	6.0	5.4	-	-	-	-	-	-	-	-	-	-	-
VII	20.6	7.7	7.0	14.6	10.0	3.7	-	-	-	-	-	-	-	-	-	-
VIII	11.6	27.9	18.3	14.4	5.5	3.4	10.2	-	-	-	-	-	-	-	-	-
IX	32.1	9.2	6.0	6.2	7.1	2.1	17.6	11.4	-	-	-	-	-	-	-	-
X	10.3	23.4	16.5	13.9	4.6	2.8	11.5	50.3	10.3	-	-	-	-	-	-	-
XI	12.4	11.8	9.4	9.4	6.1	2.1	15.2	20.0	12.5	27.2	-	-	-	-	-	-
XII	16.3	31.7	25.1	17.5	17.5	9.2	26.2	29.1	16.5	38.3	23.2	-	-	-	-	-
XIII	12.2	10.1	7.1	7.3	5.2	1.5	11.7	12.0	24.4	13.9	30.1	15.6	-	-	-	-
XIV	6.3	10.6	6.1	14.8	27.1	4.3	18.4	6.9	8.5	7.7	6.6	94.8	6.4	-	-	-
XV	9.5	32.3	19.3	41.7	84.9	9.2	13.5	17.6	9.2	17.2	11.2	24.9	7.7	16.3	-	-
XVI	7.8	7.1	6.7	8.6	14.0	3.2	29.7	6.6	18.2	7.9	7.6	27.5	8.2	20.1	9.9	-

1.1. Simulations of yeast chromosomes

1.1.1. Initial configurations used Initial configurations ($T = \infty$) disregarded confinement by the nuclear envelop, bending rigidity, anchoring of telomeres and centromeres telomere. Therefore we considered open, freely jointed equilateral chains centered at the origin of the nucleus.

1.1.2. Annealing schedule The temperature in the annealing schedule was initialized by finding the melting temperature at which the acceptance rate is just above 0.8. In our systems $T = e^1 1.0 \times k_B t$ (where kBt is the physiological value at which the distribution of the bending angles gives the persistence length reported in the article) [1]. The cooling schedule was geometric, being multiplied by a constant ($e^{-0.5} = 0.61$) at each step until finally we reached $k_B t$. At each step we performed Metropolis Monte Carlo until equilibrium was reached. Two types of moves, commonly used in polymer models, were considered in the Monte Carlo algorithm: crankshaft and random rotation arm. In the crankshaft move, two points along the chain are selected at random and the segments in between are rotated by a random angle. The random rotation arm, on the other hand selected one point and an axis of rotation and rotates the chain (from the selected point to the telomere) by a random angle.

1.1.3. Verification of final equilibrium conformations To determine whether conformations were equilibrated, we studied the convergence of the energy with respect to the number of Monte-Carlo steps at the final temperature. One expects that the mean of the distribution of energies decreases and converges exponentially to some energy value less than the initial energy value. We manually determined the number of steps $n_m anual$ for a few independent samples and selected $10xn_m anual$ the number of steps required to reach equilibrium. Convergence was validated by the theoretical distribution of external angles between chain segments for the unconfined and unconstrained worm-like chain).

2. References

[1] Kirkpatrick, S., 1984. Optimization by simulated annealing: Quantitative studies. Journal of statistical physics, 34(5-6), pp.975-986.

[2] Equilateral Random Polygon Generating Algorithm Varela R, Hinson H, Arsuaga J, Diao Y - Journal of Physics A: Mathematical and Theoretical, 2009

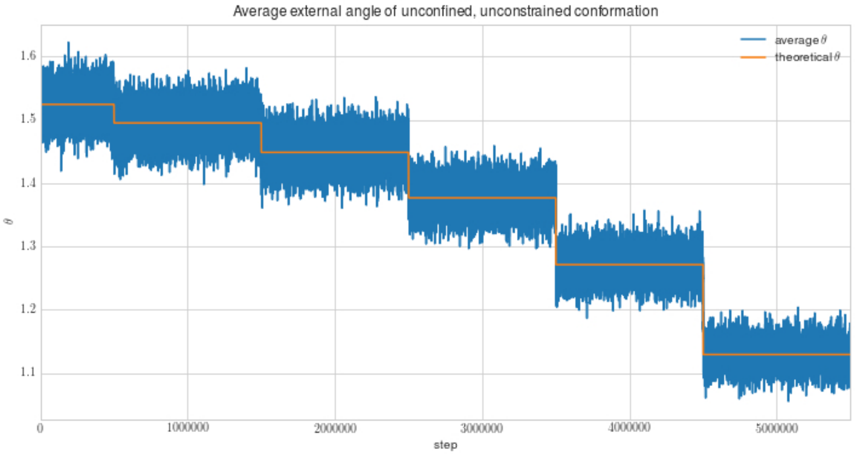

Figure S1. The average external angle $\theta_{\mathrm{sim}} = \frac{1}{n}\Sigma_i \theta_i$ of the simulated worm-like chain conformations vs. step number for unconfined, unconstrained conformations during six different steps of the cooling schedule (in blue). The energy terms are solely comprised of the WLC terms, so for each of the cooling steps the simulated values have a distribution with a mean equal to the theoretical (ensemble) average θ_{th} of the external angles (in orange).

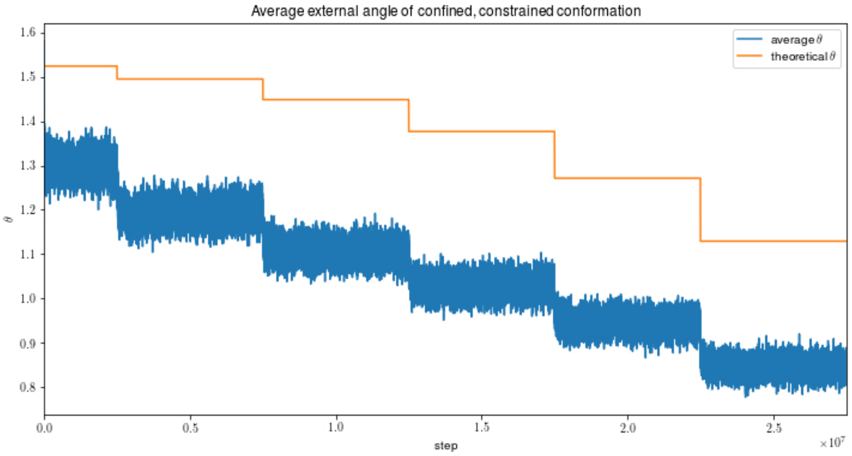

Figure S2. The average external angle $\theta_{\text{sim}} = \frac{1}{n}\Sigma_i\theta_i$ of the simulated worm-like chain conformations vs. step number for confined, constrained conformations during six different steps of the cooling schedule (in blue). The energy terms include not only those for WLC energy, but they also include terms that confine the conformation (within a radius around the center) as well as terms that constrain the certain vertices to the predetermined centromere and telomere coordinates. In this case, the simulated angle averages have a distribution with a mean less than the theoretical (ensemble) average θ_{th} of the external angles for the unconfined, unconstrained WLC (in orange).

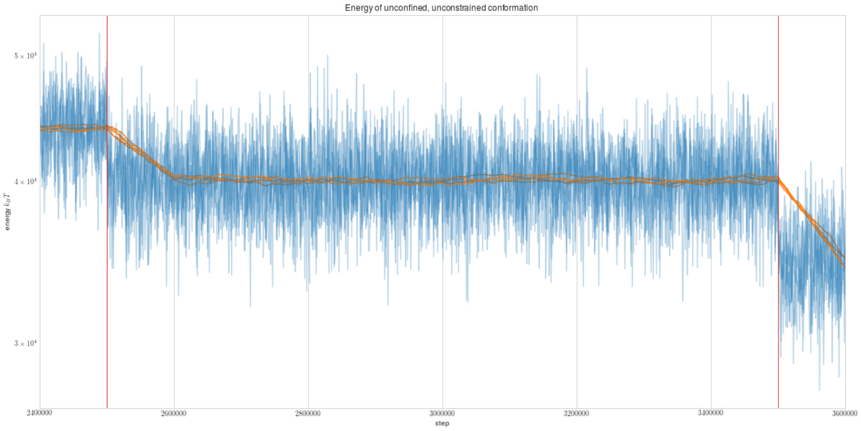

Figure S3. The energy in kBT of several different Markov-Chain Monte Carlo simulations vs. step number (blue) with rolling averages (orange) for unconfined, unconstrained conformations. The number of individual steps needed for a MCMC chain to relax within a given stage of a simulated annealing cooling schedule cannot be calculated easily as, for one, the conformation space may contain several modes, and transitions between them may be rare. Thus, by running multiple chains one can gain more confidence that a chain has in fact relaxed if all of the chains exhibit a similar distribution according to some measure (energy in this case).

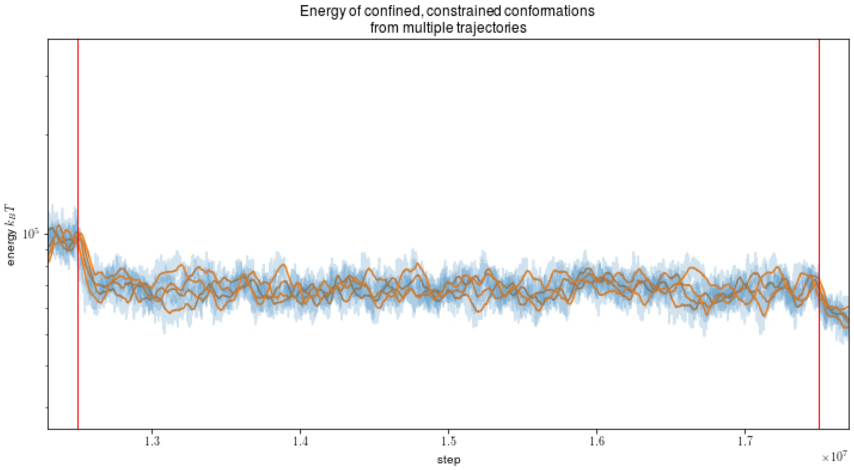

Figure S4. The energy in kBT of several different Markov-Chain Monte Carlo simulations vs. step number (blue) with rolling averages (orange) for confined, constrained conformations. Compared with the unconfined, unconstrained case, the chains take more steps to relax—as is expected, as the more complicated energy term makes the conformation space more difficult to explore using the Metroplis-Hastings algorithm.

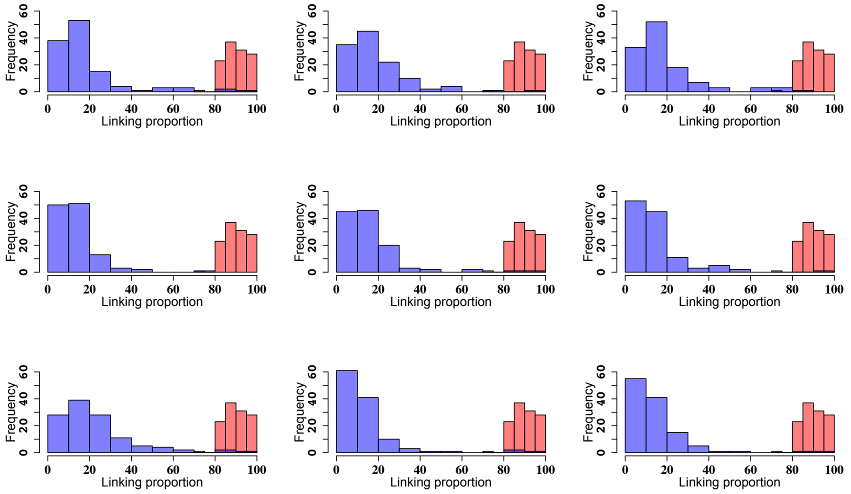

Figure S5. Histograms of linking proportions for reconstructions: 2, 3, 4 (top row), 5, 6, 7 (middle row), and 9, 11, 12 (bottom row) for randomly embedded wormlike chains. The blue histogram corresponds to the reconstructions and the red to the wormlike chains. Purple indicate overlapping values. Both models are characterized by entanglement complexity values larger than the values observed in reconstructions.

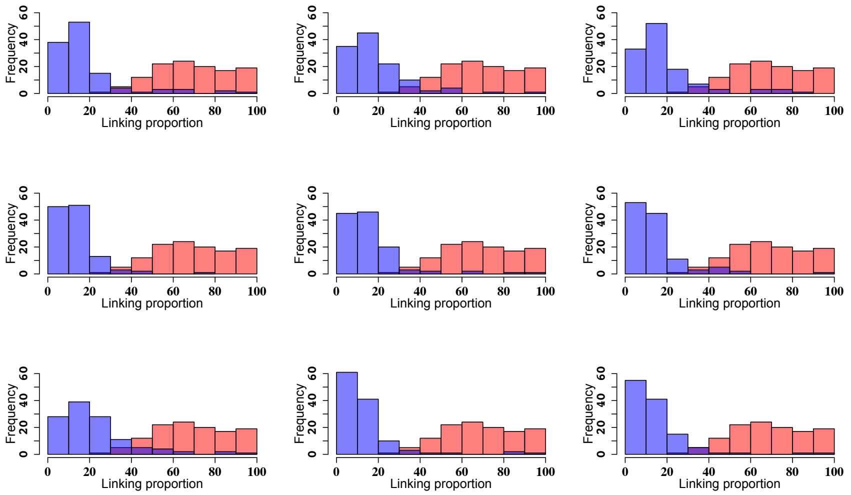

Figure S6. Histograms of linking proportions for reconstructions: 2, 3, 4 (top row), 5, 6, 7 (middle row), and 9, 11, 12 (bottom row) for randomly embedded wormlike chains with only centromeres attached to the nuclear envelope. The blue histogram corresponds to the reconstructions and the red to the wormlike chains. Purple indicate overlapping values. Both models are characterized by entanglement complexity values larger than the values observed in reconstructions.

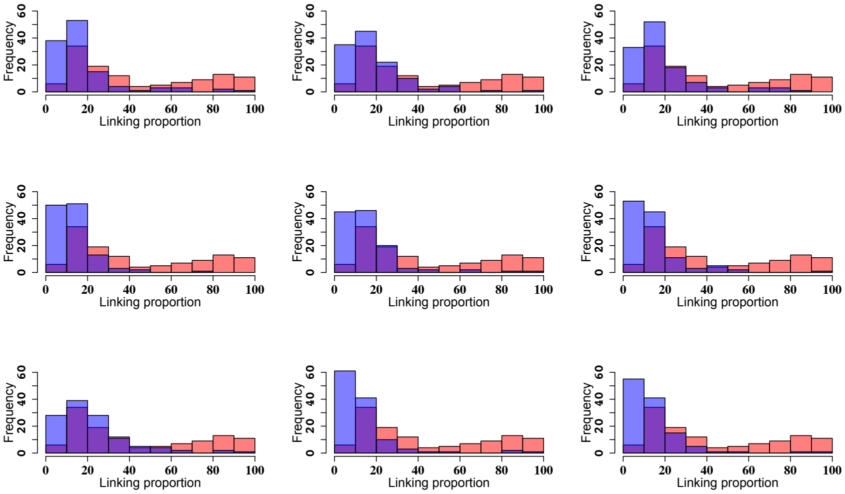

Figure S7. Histograms of linking proportions for reconstructions: 2, 3, 4 (top row), 5, 6, 7 (middle row), and 9, 11, 12 (bottom row) for randomly embedded wormlike chains with telomeres attached to the nuclear envelope. The blue histogram corresponds to the reconstructions and the red to the wormlike chains. Purple indicate overlapping values. Both models are characterized by entanglement complexity values larger than the values observed in reconstructions.

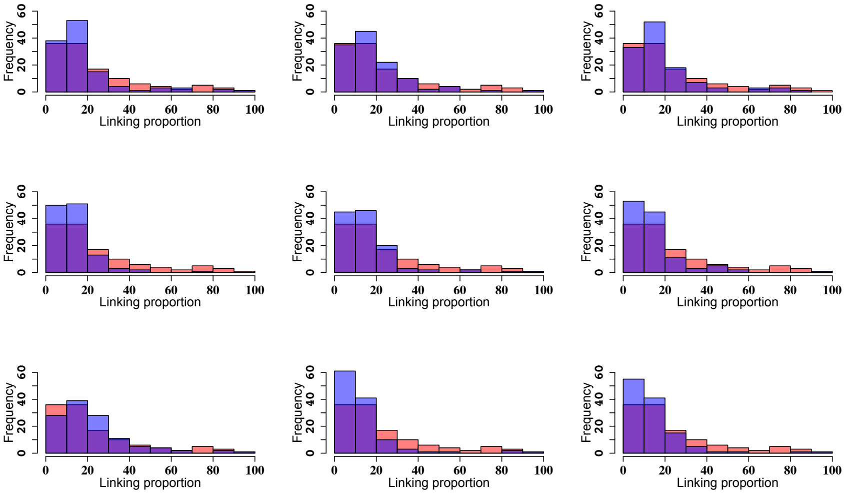

Figure S8. Histograms of linking proportions for reconstructions: 2, 3, 4 (top row), 5, 6, 7 (middle row), and 9, 11, 12 (bottom row) for randomly embedded wormlike chains with only centromeres and only telomeres attached to the nuclear envelope. The blue histogram corresponds to the reconstructions and the red to the wormlike chains. Purple indicate overlapping values. Both models are characterized by entanglement complexity values larger than the values observed in reconstructions.
